# Do site and type of metastasis in breast cancer show a changing pattern with increased age? A cross comparison of clinicopathological characteristics between age groups

**DOI:** 10.1186/s12957-018-1435-1

**Published:** 2018-07-19

**Authors:** Majid Akrami, Afrooz Sepahdar, Peyman Arasteh, Sedigheh Tahmasebi, Vahid Zangouri, Azam Askari, Babak Pezeshki, Abdolrasoul Talei

**Affiliations:** 1 Breast Diseases Research Center, Shiraz University of Medical Sciences, Shiraz, Iran; 2 Non-communicable Diseases Research Center, Fasa University of Medical Sciences, Fasa, Iran; 3 Surgical Oncology Division, General Surgery Department, Shiraz University of Medical Sciences, Shiraz, Iran; 4 Department of Internal Medicine, Fasa University of Medical Sciences, Fasa, Iran

**Keywords:** Metastasis, Age, Breast cancer, Recurrence, Pathology

## Abstract

**Background:**

In here, we evaluated pattern of metastasis and cross-compared clinicopathological features between different age groups with breast cancer (BC).

**Methods:**

This study was conducted in the Shiraz Breast Cancer Registry (largest BC registry in Iran). Patients were classified as < 30 years old (group 1), 30–60 years old (group 2), and > 60 years old (group 3). The three age groups were compared regarding clinical and baseline characteristics.

**Results:**

Overall, 564 individuals entered group 1, 4519 group 2, and 670 group 3.

Group 1 had lower rates of tumor necrosis (*p* < 0.001), higher lymphatic or vascular invasion (*p* = 0.002), estrogen receptor-negative individuals, and *HER2*-positive individuals (*p* ≤ 0.001).

Younger groups had more stage 3 BC (31.1, 25.6, and 19.7% for groups 1, 2, and 3, respectively) (*p* = 0.016), grade 3 BC (27.4, 20.6, and 16.5% for groups 1, 2, and 3, respectively) (*p* = 0.001), and grade 3 nucleus (43.1, 34.5, and 27.6% for groups 1, 2, and 3, respectively) (*p* < 0.001).

Group 1 had higher rates of regional metastasis (4.7 vs. 1.5 and 2.1% for groups 2 and 3, respectively). Younger individuals had higher rates of brain metastasis (13.3, 5.4, and 1.1% for groups 1, 2, and 3, respectively). Moreover, those > 60 years old had more lung metastasis (33 vs. 12.6 and 6.7% for groups 2 and 1, respectively) (*p* < 0.001).

Younger groups had more < 5-year recurrence (16.3, 11.7, and 8.9%, for groups 1, 2, and 3, respectively) (*p* = 0.023).

**Conclusion:**

Pattern and site of recurrence changes according to age in BC. This brings up the question whether age is an independent predictor of organ of metastasis or is site of metastasis the result of other clinicopathological determinants which differ between age groups.

## Background

An estimated 6.6%% of all breast cancers (BCs) occur in women younger than 40 years old [1]; moreover, up to 50% of all BCs are considered to occur in women over 65 years old [2, 3].

Generally, BC in the elderly population is considered to have a better clinical presentation regarding receptor status [human epidermal receptor2 (HER2) expression], grade, lymph node involvement, and tumor size [4, 5]; however, some studies have shown that older individuals are diagnosed at later stages [6]. Considering associated comorbidities and appropriate treatment of choice in the elderly population, BC’s are highly undertreated in this population, mainly due to the physician and patient’s choice [7].

On the other hand, BC’s in younger populations present another dilemma and current evidence support the idea that younger patients have overall worse prognosis, although the underlying etiology for this phenomena remains to be unclear [1, 8]. Different gene expression and BC characteristics have advocated researchers to consider the disease a separate entity among this population [9, 10].

To date, studies comparing the two populations have included limited data on patient- and disease-specific characteristics [5, 11, 12]. More importantly, multiple aspects of BC specifics remain to be addressed in these populations. Considering that previous literature has shown BC to be different between young and older patients, in here, we hypothesized that pattern of metastasis and, more importantly, location of metastasis maybe different between these groups of patients.

In an attempt to clarify differences in BC between different ages, in this study, we classified individuals in three age groups and determined clinicopathological and baseline differences between these groups in a comprehensive manner.

## Methods

### Study settings

This study was conducted in the Shiraz Breast Clinic, Shiraz, Iran which is the main referral center for breast cancers in Southern Iran. The registry is affiliated to Shiraz University of Medical Sciences and currently includes data on more than 6500 breast cancer patients, which is the largest breast cancer registry in Iran. The Shiraz Breast Cancer Registry includes data on socioeconomic status, baseline characteristics, patients’ and family clinical history, physical examination, imaging, disease course, and prognosis from every individual diagnosed with BC. The registry includes data on patients from multiple medical centers within Fars province and multiple neighboring provinces and includes a wide range of individuals from different ethnicities.

### Study protocol

According to the primary goal of the study, patients were classified according to age as followed: those under 30 years old, those between 30 and 60 years old, and those older than 60 years old. These individuals were included and classified in the age groups according to age of first diagnosis of BC. All male patients were excluded from the study.

The three age groups were compared regarding side of breast involvement (left sided or right sided); tumor size; clinical stage; treatment specifics: type of operation, hormone therapy, radiotherapy, chemotherapy, axillary management as sentinel lymph node biopsy (SLNB) and axillary node dissection (AND), and status of invasiveness of dissected lymph nodes; histopathology characteristic including tumor necrosis, in situ components, histological grade, receptor status [estrogen receptors (*ER*), progesterone receptor (*PR*), and *HER2*], pathological sub-type, and invasion status.

Stage was classified using the TNM staging system as stages 0, 1, 2, 3, and 4.

Axillary management was either SLNB, AND, both, or none.

*HER2* expression was scored according to the manufacturer’s recommendations in immunohistochemistry as followed: 0 as those with no staining or staining of membrane in 10% of cells; 1+ as weak staining in 10% of cells (cells stained in only part of membrane); 2+ was considered weak to moderate staining in all of the membrane in 10% of cells, and 3+ was considered as strong staining of whole membrane in 10% of cells.

For *HER2* expression status, patients who showed + 3 were considered positive. Those with 0 or 1+ were considered negative for *HER2* expression. For cases in which *HER2* was + 2 (or equivocal), fluorescence in situ hybridization (FISH) was performed and only individuals with a concomitant positive FISH result were considered as *HER2* positive [13].

Invasion status was considered perineural, vascular, lymphatic, two, or all.

### Statistical analysis

Data was analyzed using the SPSS software® for windows®, version 20. Patients were classified into three age groups of less than 30 years old as group 1, between 30 and 60 years old as group 2, and older than 60 years old as group 3. Comparison of normally distributed quantitative data between the three groups was done using the one ANOVA test, and for variables without a normal distribution, the Kruskall-Wallis test was used. We further used the Bonferroni post-hoc test to evaluate inter-group differences. For comparison of qualitative data between groups, the Chi-square test was utilized. For comparison of recurrence (less than 5-year recurrence and more than 5-year recurrence) between age group, those who were registered with the center less than 5 years were excluded from the analysis.

A *p* value of less or equal to 0.05 was considered statistically significant.

## Results

Overall, 564 individuals entered group 1, 4519 group 2, and 670 group 3. Comparison of baseline and clinical characteristics between age groups showed that the groups were significantly different regarding pathological sub-type, tumor necrosis, invasion status, *ER* receptor status, *HER2* expression status, BC stage, BC grade, grade of nucleus, operation type, site of metastasis, lymph node management, chemotherapy, radiotherapy, hormone therapy, and type of hormone therapy (*p* < 0.05).

Regarding BC subtypes, those over 60 years old had higher rates of mucinous type (2.2 vs. 0.9 and 0.4%), papillary type (0.9 vs. 0.2 and 0.4%), and invasive lobular carcinoma (5.3 vs. 3.5 and 0.7%) than those between 30 and 60 years old and those younger than 30 years old, respectively. Those younger than 30 years old had higher rates of mixed pattern BC (4.6 vs. 3 and 2%) than those between 30 and 60 years old and older than 60 years old, respectively; furthermore, those younger than 30 years old and those between 30 and 60 years old had higher rates of individuals with invasive ductal carcinoma (82.4 and 83.8 vs. 80.4%, respectively) (*p* < 0.001).

Evaluating tumor necrosis showed that those younger than 30 years old had lower rates of tumor necrosis than the older groups (36.4 vs. 44.6 and 48.9% for groups 2 and 3, respectively) (*p* < 0.001).

Those older than 30 years old had higher rates of lymphatic or vascular invasion; however, those in the youngest group had the highest rates of lymphovascular invasion (21.6 vs. 19 and 14.9%, for groups 2 and 3, respectively) (*p* = 0.002).

Those younger than 30 years old had higher rates of *ER*-negative individuals compared to older groups (31.2 vs. 25.5 and 23.1% for groups 2 and 3, respectively) (*p* = 0.001). *HER2*-positive individuals were significantly lower in the older age groups (19.2, 27.2, and 29.9% for groups 3, 2, and 1, respectively) (*p* < 0.001).

Individuals in the younger groups had statistically higher number of individuals with stage 3 BC (31.1, 25.6, and 19.7% for those in groups 1, 2, and 3) (*p* = 0.016) and higher rates of individuals with grade 3 BC (27.4 vs. 20.6 and 16.5% for groups 1, 2, and 3, respectively) (*p* = 0.001). This was the same with nucleus grade, as the youngest age group had the highest number of individuals with grade 3 nucleus (43.1 vs. 34.5 and 27.6% for groups 1, 2, and 3, respectively) (*p* < 0.001) (Table 1).

**Table 1:** Baseline and clinical characteristics among age groups*

Variables		Group 1 (*n* = 564)†	Group 2 (*n* = 4519)	Group 3 (*n* = 670)	*p* value
Tumor size—cm		2.70 ± 1.44	2.72 ± 1.37	2.73 ± 1.40	0.90
Breast side—no. (%)	Left	297 (52.7)	2339 (51.8)	357 (53.3)	0.727
	Right	267 (47.3)	2180 (48.2)	313 (46.7)	
Pathology subtype—no. (%)	Insitu	17 (3.1)^a^	129 (3)^a^	16 (2.5)^a^	< 0.001
	Mucinous	2 (0.4)^a^	40 (0.9)^a^	14 (2.2)^b^	
	Medullary	25 (4.6)^a^	172 (4)^a^	17 (2.7)^a^	
	Papillary	2 (0.4)^b^	8 (0.2)^b^	6 (0.9)^a^	
	Invasive ductal	448 (82.4)^b^	3650 (83.8)^b^	512 (80.4)^a^	
	Invasive lobular	4 (0.7)^a^	151 (3.5)^b^	34 (5.3)^c^	
	Mixed pattern	25 (4.6)^a^	131 (3)^b^	13 (2)^b^	
	Other	19 (3.5)^a^	72 (1.7)^b^	25 (3.9)^a^	
Insitu component—no. (%)	Yes	138 (31.1)	1058 (31.1)	161 (34.5)	0.326
	No	306 (68.9)	2339 (68.9)	305 (65.5)	
Tumor necrosis—no. (%)	Yes	169 (36.4)^a^	1536 (44.6)^b^	232 (48.9)^b^	< 0.001
	No	295 (63.6)^a^	1908 (55.4)^b^	242 (51.1)^b^	
Invasion—no. (%)	Lymphatic	49 (9.8)^a^	540 (13.7)^b^	87 (14.9)^b^	0.002
	Vascular	5 (1)^a^	80 (2)^b^	16 (2.7)^b^	
	Preneural	36 (7.2)^a^	298 (7.6)^a^	54 (9.3)^a^	
	Lymphatic & preneural	7 (1.4)^a^	109 (2.8)^a^	25 (4.3)^b^	
	Preneural & vascular	2 (0.4)^a^	37 (0.9)^a^	8 (1.4)^a^	
	Lymphatic & vascular	108 (21.6)^a^	747 (19)^a^	87 (14.9)^b^	
	None	192 (38.5)^a^	1485 (37.8)^a^	212 (36.4)^a^	
*ER* receptor—no. (%)	Positive	363 (68.6)^a^	3162 (74.4)^b^	475 (76.9)^b^	0.001
	Negative	165(31.2)^a^	1082 (25.5)^b^	143 (23.1)^b^	
*PR* receptor—no. (%)	Positive	335 (63.4)^a^	2906 (68.6)^b^	426 (69.7)^b^	0.102
	Negative	193 (36.3)^a^	1324 (31.2)^b^	184 (30.1)^b^	
*HER2* receptor—no. (%)	Positive	151 (29.9%)^a^	942 (27.2%)^a^	84 (19.2%)^b^	< 0.001
	Negative	354 (70.1%)^a^	2521 (72.8%)^a^	353 (80.8%)^b^	
Stage—no. (%)	0	17 (3.5)^a^	129 (3.3)^a^	16 (3.1)^a^	0.016
	1	101 (20.9)^a^	895 (22.9)^a^	122 (23.5)^a^	
	2	208 (43.1)^a^	1824 (46.7)^a^	272 (52.4)^b^	
	3	150 (31.1)^a^	1001 (25.6)^b^	102 (19.7)^c^	
	4	7 (1.4)^a^	59 (1.5)^a^	7 (1.3)^a^	
Grade—no. (%)	1	70 (15.9)^a^	759 (21.9)^b^	139 (28)^c^	0.001
	2	250 (56.7)^a^	1991 (57.4)^a^	275 (55.4)^a^	
	3	121 (27.4)^a^	713 (20.6)^b^	82 (16.5)^c^	
Grade of nucleus—no. (%)	1	26 (12)^a^	215 (18.3)^b^	40 (29.9)^c^	< 0.001
	2	97 (44.9)^a^	553 (47.2)^a^	57 (42.5)^a^	
	3	93 (43.1)^a^	404 (34.5)^b^	37 (27.6)^b^	

*ER* estrogen receptor, *PR* progesterone receptor, *HER2* human epidermal growth factor receptor 2

*Plus minus values are means and standard deviations unless stated otherwise. Superscript alphabets represent the results of the post-hoc test, and accordingly, different alphabets show significant difference between groups. “a” is statistically different from “b” and “c”, “b” is statistically different from “a” and “c”, and “c” is statistically different from “a” and “b”

†Age group 1 represent those younger than 30 years old, age group 2 represents those between 30 and 60 years old, and age group 3 represents those older than 60 years old

Regarding pattern and location of metastasis, those in the youngest age group had significantly higher rates of regional metastasis compared to older patients (4.7 vs. 1.5 and 2.1% for groups 2 and 3, respectively). Regarding site of metastasis, younger individuals had significantly higher rates of metastasis to the brain (13.3 vs. 5.4 and 1.1% for groups 2 and 3, respectively). Those in the younger age groups also showed higher rates of mixed pattern metastasis (more than one site of metastasis) compared to those over 60 years old (18.3 and 24.5 vs. 10.2% for groups 1, 2, and 3, respectively). Moreover, individuals older than 60 years old had higher rates of metastasis to the lung (33 vs. 12.6 and 6.7% for groups 2 and 1, respectively) (*p* < 0.001).

Regarding treatment- and prognosis-related variables, those in the older groups had higher rates of mastectomy rather than breast-conserving surgery (BCS) (57.3 vs 49.6 vs. 39% for group 3, group 2, and group 1, respectively) (*p* < 0.001).

Those in the older than 60 years old group had lower isolated SLNB managements (18.5 vs. 22.3 and 24.1% for group 2 and 1, respectively); however, these individuals had higher rates of isolated AND (73.9 vs. 67.3 and 63.6% for groups 2 and 1, respectively) and lower rates of both SLNB and AND (7.6 vs. 10.3 and 12.2% for groups 2 and 1, respectively) compared to other groups (*p* = 0.019).

Regarding number of involved lymph nodes (LNs), the three groups did not show any significant difference (*p* = 0.10).

Individuals in the older groups had significantly lower rates of radiotherapy (58.4 vs. 81.6 and 89.7% for groups 3, 2, and 1, respectively) (*p* < 0.001). Those in the oldest group had the lowest rate of chemotherapy (76.7 vs. 97 and 97.4% for groups 2 and 1, respectively) (*p* < 0.001).

Majority of those in the older groups used latrazole as their hormone therapy regimen (87 vs. 42 and 4.6% for groups 3, 2, and 1, respectively); however, majority of those in the younger groups used tamoxifen as their hormone therapy regimen (91.7 vs. 49.5 and 7.4%, for groups 1, 2, and 3 respectively) (*p* < 0.001).

Comparison of recurrence rates between age groups showed that those in younger age groups had higher rates of individuals with less than 5 years recurrence compared to those in older age groups (16.3, 11.7, and 8.9%, for groups 1, 2, and 3, respectively) (*p* = 0.023) (Table 2).

**Table 2:** Treatment-related characteristics among age groups*

Variables		Group 1 (*n* = 564)†	Group 2 (*n* = 4519)	Group 3 (*n* = 670)	*p* value
Operation type	Mastectomy	219 (39)^a^	2235 (49.6)^b^	378 (57.3)^c^	< 0.001
	Quadranectomy	342 (61)^a^	2270 (50.4)^b^	282(42.7)^c^	
Type of recurrence	Local	6 (7.1)^a^	52 (7)^a^	9 (6.2)^a^	0.338
	Regional	4 (4.7)^a^	11 (1.5)^b^	3 (2.1)^b^	
	Distant metastasis	75 (88.2)^a^	680 (91.5)^a^	133 (91.7)^a^	
Site of metastasis	Liver	8 (13.3)^a^	69 (12.5)^a^	10 (11.4)^a^	< 0.001
	Bone	21 (35)^a^	208 (37.5)^a^	28 (31.8)^a^	
	Lung	4 (6.7)^a^	70 (12.6)^a^	29 (33)^b^	
	Brain	8 (13.3)^a^	30 (5.4)^b^	1 (1.1)^b^	
	Others	8 (13.3)^a^	41 (7.4)^a^	11 (12.5)^a^	
	Mixed‡	11 (18.3)^b^	136 (24.5)^b^	9 (10.2)^a^	
LN management	SLNB	130 (24.1)^a^	957 (22.3)^a^	104 (18.5)^b^	0.019
	AND	343 (63.6)^a^	2885 (67.3)^a^	416 (73.9)^b^	
	Both	66 (12.2)^a^	443 (10.3)^a^	43 (7.6)^b^	
No. of involved LN		3.93 ± 4.93^a^	3.17 ± 3.98^a^	2.21 ± 3.35^a^	0.10
Chemotherapy	Yes	494 (97.4)^a^	3640 (97)^a^	355 (76.7)^b^	< 0.001
	No	13 (2.6)^a^	111 (3)^a^	108 (23.3)^b^	< 0.001
Radiotherapy	Yes	416 (89.7)^a^	2799 (81.6)^b^	247 (58.4)^c^	< 0.001
	No	48 (10.3)^a^	630 (18.4)^b^	176 (41.6)^c^	
Hormone therapy	Yes	327 (73.2)^a^	2746 (79)^b^	396 (86.1)^c^	< 0.001
	No	120 (26.8)^a^	729 (21)^b^	64 (13.9)^c^	
Type of hormone therapy	Tamoxifen	297 (91.7)^a^	1345 (49.5)^b^	29 (7.4)^c^	< 0.001
	Latrazole	15 (4.6)^a^	1141 (42)^b^	341 (87)^c^	
	Both	11 (3.4)^a^	208 (7.7)^b^	14 (3.6)^a^	
	Other medications	1 (0.3)^a^	24 (0.9)^a^	8 (2)^b^	
Recurrence	≤ 5 yr	42 (16.3)^a^	362 (11.7)^b^	46 (8.9)^b^	0.023
	> 5 yr	4 (1.6)^a^	100 (3.2)^a^	19 (3.7)^a^	
	no	211 (82.1)^a^	2635 (85.1)^b^	452 (87.4)^b^	

*SLNB* sentinel lymph node biopsy, *AND* axillary node dissection, *LN* lymph node

*Superscript alphabets represent the results of the post-hoc test, and accordingly, different alphabets show significant difference between groups. “a” is statistically different from “b” and “c”, “b” is statistically different from “a” and “c”, and “c” is statistically different from “a” and “b”

†Age group 1 represent those younger than 30 years old, age group 2 represents those between 30 and 60 years old, and age group 3 represents those older than 60 years old

‡Mixed are those who had more than one site of metastasis

## Discussion

In here, we compared breast cancer characteristics between three age groups to determine changes in pattern of metastasis and to compare clinicopathological characteristics between these groups.

As expected pathological sub-types were different between age groups, those who presented with BC at younger ages had higher stage, higher histopathological grade, higher grade of nucleus, higher lymphovascular invasion, higher rate of BCS, and higher rates of *HER2* expression, and majority of these individuals used tamoxifen as their hormone therapy regimen. Older individuals had higher rates of lymphatic or vascular invasion, tumor necrosis, mastectomy, axillary dissection, lower radiotherapy, and chemotherapy and mostly used latrazole as their hormone therapy regimen. As our primary outcome of study, to the best of the authors’ knowledge, we found for the first time that younger individuals had higher rates of metastasis to the brain and metastasis to more than one site (more than one organ). On the other hand, individuals older than 60 years old had higher rates of metastasis to the lung.

One of the most interesting results in our study was that although BC in younger individual is considered rare [14], we had a very high rate of individuals in the lower than 30 years old group (almost 10%), which opens a wide window for future research among these patients. This is attributed to the younger age of first presentation of BC in the Iranian population which is significantly higher than that of the Western world [15, 16].

In a recent multi-centered study by Sabiani et al. [5], pathological features of BC were compared among 5815 patients in age groups of ≤ 35 years old, 35–40 years old, 40–45 years old, and between 45 and 50 years old. They found ≤ 35-year-old patients to have higher rates of mastectomy (29.3 vs. 24.5, 21.1, and 21.1%, respectively); moreover, younger individuals had lower rates of SLNB (50.2 vs. 59.5, 65.9, and 69.8%, respectively), higher rates of AND (73.2 vs. 67.4, 63, and 57.2%, respectively), higher rates of larger than 5-cm tumors (6.1 vs. 5.2, 4.8, and 4.8%, respectively), higher rates of grade 3 BC (43.5 vs. 34.2, 25.5, and 19.6%, respectively), higher rates of lymphovascular invasion (50.6 vs. 40.9, 24.2, and 29.5%, respectively), more *HER2* positive individuals (22.1 vs. 16.3, 10.9, and 10.2%, respectively), and lower rates of negative LN (51.4 vs. 56, 58.6, and 62.1%, respectively); received less hormone therapy (52.3 vs. 60.6, 67.5, and 72.6%, respectively); had more chemotherapy (81.2 vs. 68.9, 57.5, and 50.2%, respectively); and had higher rates of distant metastasis (21.8 vs. 12.6, 9.1, and 7.7%, respectively) compared to the older groups, respectively. In our study, we found that those lower than 30 years old had higher rates of *HER2* expression; lower *ER-* and *PR*-positive individuals; higher stage and grade; higher rates of BCS, SLNB, and radiotherapy; lower rates of hormone therapy; and higher lymphovascular invasion compared to 30–60-year-old and > 60-year-old BC patients. The most important factor for the difference between the two studies relates to the different age groups (different cut-off points) which were studied, although some variables such as *HER2* expression, stage and grade, and hormone therapy show a similar trend in both studies.

In a study by Anders et al. [4], clinicopathological and prognostic determinants were compared between patients younger than 45 years old and older than 65 years old from four different cohort databases. They found that younger individuals had lower incidence of *ER* positivity, higher grades of tumor, larger tumor sizes, and higher rates of LN positivity. Their findings were very similar to our results when comparing those younger than 30 years old and those older than 60 years old.

In a cohort of 390 individuals [11], BC patient between 65 and 75 years old and individuals older than 75 years old were evaluated. They found that older individuals had larger mean tumor sizes (2.3 vs. 1.7 cm); moreover, regarding sub-types of cancer, similar to our study, in which we found mucinous sub-type to have an increasing pattern by increased age, they also found mucinous sub-type of cancer to be more frequent in their older population (8 vs. 5%). Similarly, they found a higher rate of BCS in their younger population (83 vs. 66%; *p* < 0.001); they also found lower rates of chemotherapy (4 vs. 31%; *p* < 0.001) and radiotherapy (44 vs. 67%; *p* < 0.001) among older patients. Other studies have also evaluated elderly patients, in a retrospective cohort of 317 patients from South Korea [17]; authors did not find any difference between age groups of older than 65 years old and younger than 65 years old regarding stage, nuclear grade and histological grade. However, they did find those younger than 65 years old to have higher rates of lymphovascular invasion, adjuvant chemotherapy, radiotherapy, and endocrine therapy.

Considering the different cut-offs for age groups according to different study design, an accurate comparison with mentioned studies is not possible. However, each study provides valuable information on the clinicopathological differences between different age groups. More importantly, comparing studies show some similar patterns associated with increased age, like increase in mucinous sub-type of cancer, decreases rates of *HER2* positive individuals, decreased lymphovascular invasion, and decreased grade and stage of cancers.

As the main objective of our study, we found that pattern of metastasis changes with increased age. Younger patients had higher rates of metastasis to the brain and mixed areas (more than one site of metastasis), while those older than 60 years old had higher rates of metastasis to the lung. This brings up the question whether age is an independent predictor of organ of metastasis or is site of metastasis the result of other clinicopathological determinants which differ between age groups. Among the reasons which may be attributing to the different pattern of recurrence between the age groups may be that number of individuals with triple negative BC was higher in the younger age groups, as one study showed that those with triple negative BC show higher rates of brain metastasis [18]. Although this may be attributing to these phenomena, further research is needed to evaluate the cause.

This finding may be significant in clinical practice, if proven by further research, those in younger age groups, considering their higher rates of metastasis to the brain, may require specific metastasis work-ups such as brain MRI and brain CT to rule-out metastasis to the brain which may be different from older individuals. On the other hand, older individuals with BC may need work-ups such as lung CT to rule-out metastasis to the lung, considering their higher rates of metastasis to this organ. This would significantly aid in determining prognosis and overall management pattern of BCs for clinicians.

This study was not without limitation. In here, although we included data from the largest breast cancer registry in Iran, our sample may not be representative of the whole region. We used a different cut-off for age groups in our study to compare individuals with BC and although in BC, younger than 40 is considered to be young, in the Iranian population, multiple studies have shown age of first presentation to be much younger than that of the mean global estimate [15, 16, 19]. So in this study, we categorized patients as those younger than 30 years old, 30–60, and older than 60 years old. More importantly, the different classification cut-offs do not compromise the main goal of the study which was to evaluate the overall pattern of recurrence with increased age.

Our study showed that multiple breast cancer parameters including mucinous sub-type of breast cancers, tumor necrosis, *PR* positivity, *ER* positivity, mastectomy rates, AND rates, and use of laterazole showed a higher rate among individuals in older age groups. On the other hand, parameters including lymphovascular invasion, *HER2* positivity, stage and grade of breast cancer, mastectomy rates, SLNB and both SLNB and AND rates, chemotherapy and radiotherapy use, hormone therapy, and use of tamoxifen showed lower rates in older populations.

## Conclusions

In conclusion, we found that multiple clinicopathological indexes differ in three age groups of younger than 30 years old, between 30 and 60 year olds, and older than 60 year old patients with BC. Our results showed that pattern of metastasis changes with increased age, which calls for future studies to determine the exact effect of age on mechanism of metastasis.
